# Query-Based Analysis: A Strategy for Analyzing Qualitative Data Using ChatGPT

**DOI:** 10.1177/10497323251321712

**Published:** 2025-06-06

**Authors:** David L. Morgan

**Affiliations:** 1 Department of Sociology, 6685 Portland State University, Portland, OR, USA

**Keywords:** qualitative data analysis, artificial intelligence, ChatGPT, coding

## Abstract

ChatGPT is a recently introduced artificial intelligence program that is gaining broad popularity across a number of fields, one of which is the analysis of qualitative data in health-related research. Traditionally, many forms of qualitative data have relied on a detailed process of coding the data by labeling small segments of the data, and then aggregating those codes into more meaningful themes. Instead, generative artificial intelligence programs such as ChatGPT can reverse this process by developing themes at the beginning of the analysis process and then refining them further. This article presents a specific three-step process, query-based analysis, for using ChatGPT in qualitative data analysis. The first step is to ask broad, unstructured queries; the second is to follow up with more specific queries; and the third is to examine the supporting data. A demonstration of this process applies query-based analysis of an empirical dataset that consists of six focus groups with caregivers for a family member experiencing cognitive impairment, who discussed their experiences in seeking diagnosis for their family member. The conclusions consider the potential impacts of query-based analysis on traditional approaches based on the coding of qualitative data.

## Introduction

ChatGPT is a generative artificial intelligence (AI) program that was introduced in the fall of 2022 and has shown immediate and widespread utility in a range of fields. This article continues that trend by demonstrating the value of ChatGPT for the analysis of qualitative data, with an illustrative example from health-related research. Although the potential value of generative AI in this regard has been examined (Christou, 2023; Friese, 2023; Hamilton et al., 2023; Hitch, 2024; Morgan, 2023; Silver, 2023; Wachinger et al., 2024), there have been few systematic descriptions of step-by-step procedures for using this program as a general approach to producing themes in qualitative data. This article presents such a framework.

The article begins by explaining the relevance of ChatGPT to qualitative data analysis, and a comparison to coding as a more traditional approach to that field. It then lays out a three-step set of procedures, called query-based analysis (QBA), for analyzing qualitative data using generative AI overall and ChatGPT in particular. This three-step process is illustrated with a worked example involving focus groups of caregivers of people with Alzheimer’s disease. The article concludes with a consideration of the possible impacts of AI on the field of qualitative data analysis.

## Background

This article follows a previous one (Morgan, 2023) that provided a “proof of concept” for the use of ChatGPT in qualitative data analysis. That earlier article demonstrated the power of ChatGPT for summarizing large amounts of qualitative data in two datasets, and argued for using an iterative, conversational approach between the researcher and the AI. The goal of the present article is to move beyond that earlier, broad presentation to develop a specific technique that applies ChatGPT to the analysis of qualitative data.

### ChatGPT and Traditional Software for Qualitative Data Analysis

Various versions of Computer-Assisted Qualitative Data Analysis Software (CAQDAS) have been available for over 30 years, but note that the emphasis is on “computer-assisted,” because these programs do not perform the analysis for you. Instead, their origins are in the computerization of traditional manual coding, which relies on attaching labels to small segments of text, then collecting those more specific codes into broader categories, and ultimately creating more meaningful interpretations. This process of working from observations to a set of interpretive themes is widely accepted as inductive. At present, the state of the art for CAQDAS programs is the continuing addition of new features that facilitate the use of codes in the more interpretive portion of the analysis process.

It should be clear that coding and the search for themes are but one approach to qualitative data analysis. For example, Freeman (2016) lists this kind of thinking as based on categorization, and presents four other alternatives. Still, coding represents the dominant trend in qualitative data software, and this has not changed in more than 30 years (Tesch, 1990). In contrast, my proposed version of QBA begins by asking the AI program broad questions about the data, and then working with the responses the AI provides to generate more interpretive content. The first set of queries in QBA simply asks ChatGPT or an equivalent program to summarize the data. Note that semantically, the broader field of AI typically refers to “prompts” rather than “queries,” but I have chosen the latter term to emphasize the question-and-answer process in QBA.

As with coding, QBA’s process of moving from general summaries of observations to more interpretive conclusions follows an inductive path. In addition, both coding and QBA bypass the need to pre-specify themes, which would be necessary for a deductive approach. However, coding and QBA implement their shared preference for induction in very different ways. For coding, this amounts to fracturing the data into many small codes, and then progressively reassembling it into a few broader themes. In contrast, QBA reaches such themes through an extended conversation with the data through AI.

Silver (2023) describes three options for incorporating AI into qualitative data analysis: first, to use it alongside existing approaches to qualitative data; second, to build it into existing software for the analysis of qualitative data; and third, to rely on it alone as an analysis tool. For now, the state of the art is represented by her second option: incorporating AI into existing software. Early on, NVivo introduced tools based on what is known as natural language processing, a way to produce “auto” coding (Lumivero, 2023b), and sentiment analysis (Lumivero, 2023a). More recently, ATLAS.ti (2023) and MAXQDA (2023) have incorporated access to ChatGPT into their programs, but as extensions of existing CAQDAS, both of these implementations are based on an assumption that qualitative data analysis consists primarily of coding, with AI as an assistant to that process.

QBA corresponds to Silver’s third option by utilizing an AI as the sole analytic tool. Developing QBA as a stand-alone method for qualitative analysis requires a systematic specification for applying generative AI programs such as ChatGPT to qualitative data, so this article will develop an explicit, three-step version of QBA, which serves as an alternative to coding. In doing so, I treat both coding and AI-focused analyses as tools that are not inherently limited to any distinct theoretical orientation. Instead, it is up to the researchers in a given field to determine the relevance and effectiveness of AI for their goals (van Manen, 2023), just as they would any other proposed method. In the present case, QBA moves from treating AI as a generic tool to developing a specific method for employing that tool, which the next section will demonstrate.

## The Empirical Example

The research project that will be analyzed with ChatGPT involved seeking diagnosis for a cognitively impaired family member (see Morgan, 2002 for a complete description of this study). Because the basic nature of Alzheimer’s disease and other forms of dementia reduces the patient’s own self-awareness, this places responsibility on family members to determine the meaning of the symptoms that they perceive. In particular, as dementia progresses, the family is increasingly likely to seek a formal diagnosis to explain the changes they are observing. The ethical approval for this study was obtained from the Human Subjects Review Board at Portland State University. In this case, the analysis was done on what amount to secondary data, which were collected well before the present work was done. The data were fully anonymized, and ChatGPT was set to not upload this data for its future reference database. Taken together, these steps ensure the participants’ privacy.

Our research team began by locating participants through an expert diagnostic clinic. We received permission from a human subjects review process to contact the family members of clinic patients who were diagnosed with dementia. We asked those family members who had a decision-making role in seeking the diagnosis, and then invited those involved in that decision to participate in focus groups. In addition, we used the clinic’s diagnostic testing to divide our cases according to whether the patient had either less severe or more severe symptoms at the time of diagnosis.

We chose focus groups as a data collection method because they are especially useful for hearing how participants share and compare their experiences about decision making (Morgan, 2019). Further, the focus groups allowed us to examine consensus and diversity in caregivers’ experiences. These goals were aided by a division between groups according to whether families sought diagnosis in the presence of either less or more severe symptoms. From the participants’ point of view, this separation ensured that they were talking to others who made their decisions at a similar point in the development of the illness. From a researcher’s perspective, this allowed us to gain detailed information across the full progression of the caregivers’ decision-making processes.

The total dataset was thus divided into two segments, with three groups where the clinic’s testing indicated that the patients had less severe symptoms and three groups where the patients had more severe symptoms. There were six family caregivers in each group, and the typical interview lasted about 90 minutes generating a transcript that was approximately 15,000 words in length. The same questions were asked in all six groups, to ensure comparability across the full dataset.

The moderator’s interview guide consisted of six questions organized around taking a history of how each family decided to get a diagnosis. The first set of questions asked about the caregivers’ perceptions of the earliest symptoms, how family members shared this information among themselves and others, and changes in symptoms over time. The second set asked about the decision to contact a doctor or other health professional, as well as how the family chose this specific hospital for an expert diagnosis. The final question asked the participants about the advice they would give to other families facing similar decisions.

In terms of analysis, my earlier work with this data concentrated on the differences between these two groups of caregivers (Morgan, 2002), so the emphasis in the present investigation was on the experiences and feelings that these caregivers shared in common. Because there was no intentional difference across the groups, other than the severity of symptoms at the time of diagnosis, I investigated that dataset as a whole, rather than as a set of six separate cases. Importantly, my prior analysis gave me a detailed familiarity with the data based on the mutual experiences, thoughts, and feelings across all the caregivers versus what separated the two subgroups.

The research project was approved by the Institutional Review Board at (Portland State University), and the data were fully anonymized prior to analysis. In addition, I activated the option in ChatGPT not to include this data in its future training set.

The actual dataset for this analysis consisted of a single PDF file that combined all six interviews, because the goal was to look for shared themes, rather than to compare the content of the separate groups. In addition, I removed the moderator’s questions and any probes that recapitulated the answers to those questions. This decision to not include the moderators’ remarks follows the traditional procedures of not coding anything that the interviewer’s says, but more importantly for AI-based analyses, it ensures that the program will not include the topics introduced by the interviewer in the program’s interpretation of the data. Eliminating the moderator’s content makes sure that both the analyses and the conclusions from them relied solely on the comments by participants. And in this case, since I served as the interviewer for all six focus groups, I was able to ensure that all responses from ChatGPT were interpreted in the proper context.

## Applying ChatGPT to Qualitative Data Analysis

At the time I conducted these analyses, it was not possible to input a dataset this large directly into ChatGPT. Consequently, I compared two different “pre-processor” programs that performed this function, ChatDOC (ChatDOC, 2023) and ChatPDF (ChatPDF, 2023). I also repeated the analyses using ChatGPT 3.5 and ChatGPT 4.5. Ultimately, all of these combinations returned similar results, so I chose a pairing of ChatDOC and ChatGPT 3.5, based on their features for the present analysis. Specifically, as the presentation in Step 3 in the QBA process will illustrate, this was the only combination that made it possible to mark relevant content in the data itself. In addition, the presence of such coding throughout the data suggests that there were few if any problems with the size of the dataset (i.e., “token limits”).

In terms of settings for ChatGPT 3.5, there is an option to prevent the program from assimilating the current dataset into its future training data, and I activated this option to help preserve the participants’ privacy. In addition, the program has a setting for “temperature,” which controls the randomness or creativity of its responses; this ranges from 0 to 10, and I set it to zero to assure the accuracy of the responses to my queries.

### Step 1: Asking Broad, Undirected Queries

My recommended strategy for applying QBA is organized around three basic steps, the first of which is to ask broad, undirected queries. The goal at this initial stage is to locate a set of basic topics, or themes in the data that can serve as the foundation for further searching. Note that this starting place does require a reasonable degree of familiarity with the data, which would typically result from having a meaningful role in collecting the data. The most desirable form of familiarity would result from both collecting the data oneself and then re-reading the transcripts. When that degree of familiarity is not the case, then a more intense reading of a substantial sample of the data should occur prior to any querying. However one achieves the necessary familiarity with the data, it is essential to take a reflexive stance toward the decisions being made throughout the analysis process. This need for a conscious degree of reflexivity is especially important for QBA, given its reliance on the researcher’s subjective judgments in interpreting the responses from the AI.

The wording for a typical first query begins by setting a context for the dataset as a whole, such as: “The individuals who participated in these interviews were [description] and they discussed [topic] …” This statement would be accompanied by the query itself, such as: “What were the key topics in this document?” By requesting such a generic reply, QBA takes a fundamentally inductive stance, rather than supplying any prior theory or predetermined content that would be necessary for a deductive approach. Instead, by asking for the most general content first, it works from the bottom-up to more interpretive conclusions. This is matched in ChatGPT by providing broad summaries as a starting point, before responding to more targeted requests. Ultimately, however, it is how the researcher uses the content produced by the program that determines the inductive nature of the QBA as a form of data analysis.

Accompanying this inductive approach is a subjective judgment of the results from this querying process, and the standard for evaluating the AI’s initial response should be the extent to which that answer captures the original research goals. If it is too far off those goals, then the most likely solution is to adjust the context statement that you initially supplied, possibly by adding a one-sentence summary of the research question. Note that with ChatGPT, it is not necessary to supply this context with every subsequent question in a series because the program will remember it without further querying.

Once you have established that the QBA can match the research goals, then it is important to recognize that no one query is likely to capture a well-formed list of candidate themes. Although repeating the same query to ChatGPT can produce some differences in the responses, I recommend explicating varying the queries that you use in this initial step. Other examples of querying at this stage would include: “What are some of the main themes with regard to [research topic]?” Or, “Give me a list of the things that mattered most to these participants.” Or, “Give me a long list of the factors that affected how these participants …” In comparing these questions, one key difference is whether you explicitly request that the program give you a list of the items that make up the content you are seeking. In my experience, it is unpredictable whether a program will return a narrative description or list (which may be either numbered or bulleted), unless you specifically state the format you want. Since the purpose in this step is to decide on a set of core themes that can serve as the basis for further queries, some form of list is almost always the preferred response.

#### Demonstration of Broad, Undirected Querying

I began by setting a context for the present dataset: “This data comes from focus groups where family caregivers discussed seeking diagnosis for a family member with Alzheimer’s Disease.” After that, I experimented with three different sets of initial queries, as summarized below. After each of these three queries, I used the program option to “reset” or “clear” the previous conversation, so that none of the subsequent responses were affected by any of the prior queries. I then assessed each of these lists of themes, and thus the strategy that produced it, according to my knowledge of these data. Note that the three alternate wordings for the query are essentially an example of “prompt engineering,” with the goal of determining the extent of the shifts in responses as a function of the phrasing of the prompts.

• Querying for the basic research question, for example, “Give me a list of the key themes that affected when and why these caregivers sought diagnosis.”
• Querying for a more detailed version of the larger research goals, for example, “Give me a list of the factors that led some of these caregivers to seek diagnosis earlier when the symptoms were relatively mild” and “Give me a list of the factors that led some of these caregivers to seek diagnosis later when the symptoms were relatively severe,” followed by: “Combine the two previous searches to give me a list of the key themes that affected when and why these caregivers sought a diagnosis.”
• Querying for a summary of the responses to each of the six original interview questions, one by one, as suggested by Kuckartz and Radiker (2023), for example, beginning with, “Tell me about the caregivers’ perceptions of the earliest symptoms,” and so on and each of the other five questions, followed by: “Combine the six previous searches to give me a list of the key themes that affected when and why these caregivers sought a diagnosis.”

The thematic outcomes of these searches are shown in Table 1. All three outcomes show an essential similarity. Comparing these sets of themes, I judged the best summary to come from the query based on the six original interview questions, which produced a compact list of the most important elements in the data. The one exception was its final theme on financial considerations, which I judged to be more of a “topic summary” (Braun & Clarke, 2022), rather than a truly interpretive theme. In particular, my familiarity with the data indicated that although discussions of finances certainly did occasionally occur, they failed to spark anything like the same level of active discussion as the other five candidate themes. Overall, the similarity of the three sets of responses reinforced my confidence in the analysis outcome, indicating that it would have made little difference which of the three sets of candidates I pursued.

**Table 1.** Results From Three Basic Searches for Themes.

Basic Themes	Research Questions	Interview Questions
Concerns and observations	Need for a diagnosis and information	Need for a specific diagnosis
Progressive decline	Emotional and mental well-being	Desire for information and coping strategies
Need for support	Concern for the safety and well-being of the person with Alzheimer’s	Lack of communication from healthcare professionals
Family involvement	Seeking expert guidance and support	Impact on caregivers’ sanity and well-being
Professional recommendations	Desire for financial and legal planning	Planning and organizing for the future
Knowledge and awareness	Recommendations from others	Financial considerations

Both my selection of the themes based on the questions from the interview and my decision to drop financial considerations as a theme illustrate a fundamental point about QBA: Nothing can replace the researcher’s substantive judgments about the most important elements of the analysis. In particular, you need to make your own decisions, and to feel confident that you can justify those decisions.

Hence, the key goal in this first step is not to devise a single, perfect query that eliminates the researcher’s judgment. Instead, the reason for consulting a series of queries is to produce what one judges to be best set of themes. Within the broader field of AI, successively comparing the results of different queries is known as “prompt engineering” (Chubb, 2023; White et al., 2023). It is also important to note, however, that the assessment of which prompts produce the best results is inherently subjective, so it is crucial that reports of the analysis process should include, whenever possible, the actual wording of the prompts that were used. Overall, my recommendation is to engage in prompt engineering by comparing the responses to different prompts, and then rely on your familiarity with the data to select your preferred themes, based on their ability to capture what is most meaningful in the data. Once again, this is a reflexive process that requires researcher to engage in subjective judgments, based on a strong familiarity with the data to be analyzed.

### Step 2: Following Up With More Specific Queries

The goal in Step 2 of QBA is to use a series of more specific queries to generate a more detailed elaboration on the basic themes that you located in Step 1. In undertaking this stage, it is important to execute it as a continuation of the queries that you used in Step 1. Once again, this is because ChatGPT updates the depth of its understanding of the data with each successive query, so that the responses to these new, more specific queries will take into account the answers to the earlier set. Explicitly building on the results from Step 1 avoids any implication that the software itself is capable of generating subthemes, and instead emphasizes the need for hands-on inputs from the human analyst. Instead, this pursuit of more complex analyses needs to be custom tailored to the research goals at hand.

One likely way to proceed is by asking about each of the themes that made up the results in Step 1. In essence, this is equivalent to seeking subcategories under the major themes generated earlier. Note, however, that it is unlikely that each subtheme will be found once and only once under each of the original themes; instead, versions of the same subtheme will often appear under more than one of those original themes. This makes sense because the original themes are almost certain to be related to each other, and the presence of similar subthemes across the themes reflects these relationships. This process of delving more deeply into the relations among the basic themes goes beyond typical approaches to thematic analysis.

When there are multiple overlapping subthemes, the next activity in Step 2 of QBA is typically to reduce the total number of subthemes. This process of comparing themes and subthemes helps to “thicken” the analysis, as opposed to reporting an exhaustive list of all the subthemes generated by the AI. ChatGPT facilitates this process by producing not only a title for each subtheme but also an explanation of its content, so it is relatively straightforward to make comparisons across the full set of subthemes.

#### Demonstration of More Specific Querying

In the empirical example, I began this step by examining the equivalent query for each of the five basic themes, as illustrated for the first theme. This is once again an example of prompt engineering, which would be open to further experimentation in future work. (Note that the numbers included with each response will be explained in Step 3).

The query was, “Give me a list that tells me more about how the earlier theme of [need for a specific diagnosis] affected when and why these caregivers sought a diagnosis.”

ChatGPT produces the following response: The themes of the “need for a specific diagnosis” affected when and why these caregivers sought a diagnosis in the following ways:

1. Desire for a clear diagnosis: Many caregivers expressed a desire for a specific diagnosis, such as Alzheimer’s disease, rather than a general term like dementia [**23**]. They felt that having a clear diagnosis would provide them with a better understanding of the condition and enable them to plan and organize their lives accordingly [**140**]. This need for a definitive diagnosis drove them to seek medical evaluation.
2. Emotional response to diagnosis: Receiving a diagnosis was an emotional experience for caregivers, with some individuals expressing surprise and sobbing when Alzheimer’s disease was officially diagnosed [**140**]. The diagnosis helped them come to terms with the condition and move forward with care plans and support.
3. Access to information: Caregivers mentioned that a specific diagnosis helped them gain access to valuable information about the disease, including its symptoms and ways to cope with the changes it brings [**92a**]. They wanted to understand the specific symptoms and behaviors associated with Alzheimer’s disease to better support their loved ones and manage the challenges they would likely face.
4. Planning and preparation: The need for a specific diagnosis was linked to the caregivers’ desire to anticipate and be prepared for the changes and progression of the disease [**98a**]. With a clear diagnosis, they could make informed decisions regarding safety, such as preventing their loved ones from driving and ensuring they received proper care and support [**98a**].
5. Validation and support: Obtaining a specific diagnosis provided caregivers with a sense of validation and relief. It helped them validate their concerns and convictions about their loved ones’ condition, especially when seeking second opinions from reputable clinics like (Local Hospital) and the (National Hospital) [**71**][**98b**]. They also found comfort in joining support groups, where they could connect with others who shared similar experiences and learn from their insights [**92b**].

These factors collectively influenced caregivers to seek a specific diagnosis earlier in the disease progression, allowing them to better understand and care for their loved ones with Alzheimer’s disease.

The response to the query for this first major theme shows five subthemes. In total, the searches for the full set of five original themes produced 25 possible subthemes. To deal with analytical complexity, I made comparisons of the contents of those candidates for subthemes using the full text descriptions for each. Table 2 shows the final list of subthemes after consolidating the overlapping entries.

**Table 2.** Reduced List of Candidate Subthemes.

Need for a specific diagnosis
1. Desire for a clear and timely diagnosis (2)
2. Emotional response to diagnosis (1)
3. Anticipating changes (5)
4. Planning and organizing (3)
5. Need for support (5)
Desire for information and coping strategies
1. Anticipating changes (5)
2. Self-education and seeking advice (2)
3. Dissatisfaction with doctors (3)
4. Planning and organizing (3)
5. Need for support (5)
Lack of communication from healthcare professionals
1. Desire for a clear and timely diagnosis (2)
2. Anticipating changes (5)
3. Self-education and seeking advice (2)
4. Dissatisfaction with doctors (3)
5. Need for support (5)
6. Lack of referrals and recommendations (1)
Impact on caregivers’ sanity and well-being
1. Emotional strain and personal sanity (1)
2. Anticipating changes (5)
3. Dissatisfaction with doctors (3)
4. Need for support (5)
Planning and organizing
1. Financial considerations (1)
2. Anticipating changes (5)
3. Safety concerns (1)
4. Need for support (5)

Table 2 also shows notations in parentheses for how many times versions of each subtheme occurred. Allowing for these overlaps shows a total of 10 unique subthemes, two of which, “Anticipating changes” and “Need for support,” occurred in all five of the major themes. In addition, there were no subthemes that occurred four times, two that occurred three times, another two that occurred two times, and four that occurred only once. Consequently, I decided on a final set of results that consisted of the five major themes, with each of them containing the two subthemes that appeared throughout.

The pattern of overlaps found in Step 2 of QBA represents an important resource for further refinement of the basic themes. The method used in this example represents only one technique for working with these overlaps. Another advanced analysis technique would be to use network diagrams or concept modelling (mind mapping) to link the core themes into higher-level models. For now, however, the next basic step in QBA is to assess the connections between each candidate theme and the original data that support it.

### Step 3: Examining the Supporting Data

The goal in this portion of QBA is to substantiate the basic themes and subthemes derived from the earlier analysis. More specifically, Step 3 examines the text to select quotations for inclusion in the Results section of the research report. This follows a traditional pattern in reporting qualitative research where the Results section presents a set of themes, each of which is illustrated by a set of quotations from the original dataset. This requires linking the candidate themes from Step 2 to their underlying data, and some programs, such as ChatDOC, provide these links as part of their responses. For other programs, you may need to generate specific queries to retrieve this information (e.g., “Give me direct quotations from the data that are related to …”), and then be careful to search the data itself to validate the suggested questions that the AI returns.

#### Demonstration of Selecting Quotations

One of the reasons I used the ChatDOC preprocessor program for the current analysis is because it automatically returns links between its summaries and the sources in the data for those summaries. These links appear as bold face numbers at the end of each summary, as shown earlier, which contains eight recommended connections to the text for the theme “need for a specific diagnosis.” For example, the quotable material at site [**23**] in the data consists of:

I was hoping for a diagnosis. I was hoping for another word besides dementia which my parents, they just absolutely had troubles with that, and I just felt like getting the truth out and going on from that point, whatever that might be.

As noted earlier, another option is to use the AI as a tool for querying about quotable text segments. For this purpose, I chose to use subthemes, and I wanted to ask for quotations that combined aspects of the results from Stage 2, such as: “Give me direct quotations from the data that are related to both the need for a specific diagnosis and anticipating changes.”

ChatGPT responded with several quotations, beginning with:

I wanted to rule out anything else and probably I wanted information as to what are some of the specific symptoms. I think people in general need to know that. More what are the symptoms, what are some of the things that you can do to help deal with those changes that take place in your family member’s life.

In both of these examples, I have simply chosen the first quotation suggested by the AI to demonstrate the searching process. A complete data analysis would involve searching all the proposed quotations to select the ones that were most effective for conveying the underlying content of each theme. Even so, this illustrative version of Step 3 shows how ChatGPT can search through a complex text document to locate the desired kind of supportive material.

Overall, the full set of examples from all three steps in applying QBA to this empirical study help establish the effectiveness of ChatGPT as a tool for qualitative data analysis. More specially, these examples demonstrate how the three-step process in QBA moves from the raw data to the basic components of the Results section in the substantive analysis of a real dataset.

## Discussion and Conclusions

Before considering the advantages of both ChatGPT as a general tool for qualitative data analysis and QBA as a means for implementing that tool, it is important to consider some of the limitations of the current presentation. First, there are ethical issues, some which are continuous with previous versions of qualitative research and some of which are new to AI-based analysis, and the most important concern in both cases is the need to maintain participants’ privacy. In each instance, a keystone provision is the need to deidentify (“anonymize”) the data as thoroughly as possible, and this should be done either during transcription or in an early review of the transcribed data. Where the use of tools such as ChatGPT raises new issues is with regard to whether the data will be shared outside the research team, through incorporation into the AI’s “training set” (i.e., the data that generative AI’s use as part of their learning process). Fortunately, it is possible to turn off this option in ChatGPT 3.5, as I did in the present analysis, so that the data being analyzed remains private. This combination of deidentified data that are examined without inclusion in the AI’s capabilities should equal the current standards for maintaining privacy in qualitative data analysis.

A second issue, which in this case is unique to AI-based analysis, is that ChatGPT has been found to contain biases and to produce “hallucinations” when working with data from the internet. In working with two previous datasets (Morgan, 2023) as well as the present one, I have not detected any overt instances of either bias or hallucination, but of course this is not enough to rule out the wider possibility of such problems. Hallucinations are often conceived as outright, factual error, and Wachinger et al. (2024) report that when they asked ChatGPT for examples of a specific theme, they received some non-existent quotes, while Deiner et al., 2024), in a systematic counting, found such hallucination to be quite rare, but still present. I thus recommend asking for quotes in an express format (“Give me direct quotations from the data that are related to …”), and then double-checking them against the data itself. Of course, there is also the possibility of larger scale hallucinations at the level of the themes themselves, but I am not aware of any reports of such problems.

With regard to bias, its source lies in the training set of data that is the basis for any generative AI. To the extent that this original input was biased with regard to factors such as race or gender, this can be carried over into the analysis of user-supplied data. In the present case, a discussion of how this has been handled in ChatGPT can be found at ChatGPT (2023), which details their work at minimizing the problem of bias. Still, for researchers who work with topics that may be sensitive to misrepresentation or under-representation in the general population, there is once again no substitute for checking the output of the AI through one’s own familiarity with the data. In the end, however, there is no guaranteed system for detecting and eliminating bias in AI-based analysis.

Moving beyond known faults in AI, a more general limitation is the restricted range of existing applications of AI to qualitative data analysis. In essence, we are still in a highly exploratory phase for discovering both the strengths and weaknesses of this tool. My argument here is that specifying techniques for implementing such analyses will help build the kind of practice-based experience that allows us to assess those strengths and weaknesses.

One specific element of this further development is the extent to which AI-based analyses should include a reliance on the researcher’s subjective judgments. My position is that QBA must necessarily keep a “human in the loop” to both question and interpret the responses from the AI. Once again, this requires considerable familiarity with one’s data. There is no way that one can simply feed an unknown dataset into a program and expect it to do the analysis for you. Instead, there is a continual set of decisions that require a reflexive stance toward the data at hand.

This reliance on human judgment is essential for judging the validity of any theme-based analysis, which Braun and Clarke (2022) refer to as “reviewing themes” in Step 5 of their six-step process. More specifically, the validation of themes must address two different questions: first whether the proposed themes are indeed important aspects of the data (a problem with “false positives”) and second whether any important themes have been omitted (a problem with “false negatives”). For false positives, if there are relatively few sections of the data that match a potential theme, then it may not be sufficient for inclusion in the full set of themes. This is traditionally assessed by searching for an adequate set of appropriate quotations, as in the third stage of QBA.

For false negatives, one common way of protecting against this error is through deep familiarity with the data, which is the path I followed in the previous illustrative analysis. Whenever that familiarity is not available or a more rigorous approach is necessary, an alternative way to ensure the rigor of the thematic outcomes is what I have called (Morgan & Nica, 2020) *evaluating* candidate themes using traditional software. This requires systematically applying the set of potential themes to the original data, which allows an assessment of both whether each theme is well developed in the data and whether there are any meaningful topics in the data that were not included in these themes. Note, however, that such a thorough approach is only rarely needed, and even when it is used, the inclusion of any given theme in the final set still requires a subjective judgment about whether there is sufficient related content to justify its selection.

A different approach to validation can be found in AI-based analyses that have used those programs for some version of automated coding as a way to produce themes (e.g., Dengel et al., 2023; De Paoli, 2023, 2024; Heseltine & Clemm von Hohenberg, 2024; Jalai & Akhavan, 2024; Lee et al., 2024; Prescott et al., 2024; Turobov et al., 2024; Wachinger et al., 2024; Zhang et al., 2023, 2024). The emerging quality control standard in that literature is to compare machine-produced themes with those from manual coding of the same data, to determine whether the two are reasonably comparable. An equivalent approach could be used to generate head-to-head comparisons between QBA and traditional methods of analysis. This would require a somewhat different testing procedure, however, because of QBA’s insistence on keeping a human in the loop by consistently relying on the researcher’s subjective judgments, rather than attempting a complete mechanization of the analysis process. So, the same person could not independently produce the two sets of results. This could be resolved by having two separate persons or teams each do their own analysis, one using manual coding and thematizing and the other using a theme-based AI-approach such as QBA. Although no one such test can be conclusive, the current emphasis on such comparisons in the literature would provide a particularly useful direction for future research that assesses QBA on the same standard.

Of course, any attempt at validation can be controversial in qualitative research, and this is especially so when the goal is to produce themes. Thus, Braun and Clarke (2022), Charmaz (2014), and Smith et al. (2022) all explicitly argue that searching for themes is an inherently subjective process that cannot be treated as if there is only one, valid, explanation for the data. For research based on generative AI, as I argued earlier, even small shifts in the queries that are posed to ChatGPT can lead to notable changes in the responses. Further, submitting the identical query to other programs, such as Claude or Gemini, is almost certain to produce difference responses. This degree of unpredictability requires resisting the temptation to treat any program’s responses as a definitive, objective interpretation of the data.

Returning to the comparison between AI-based analysis and traditional forms of coding, another clear concern is whether relying on AI for qualitative analysis creates too much “distance” between the researcher and the primary data. Of course, any kind of coding can become mechanical, but in general there is no denying that detailed manual coding immerses the analyst in the data more than AI-based approaches. So, once again, there is no substitute for a familiarity with one’s data and a reflexive approach to one’s decision making.

A further comparison between traditional forms of coding and beginning the analysis with an AI-based summary points to eliminating the fundamental processes involved in coding data, that is, the labeling of small data segments and the progressive aggregation of those codes into more meaningful categories. This is by far the most time-consuming element in coding as a method for qualitative analysis, so replacing it is a valuable accomplishment in and of itself. In addition, relying on generative AIs such as ChatGPT minimizes the need for specialized software that can be challenging to master. I personally am proficient with three different CAQDAS programs, and I can testify that it took less than 2 hours to master ChatGPT, as opposed to several days for each of the traditional programs—but this is only one case.

This comparison to coding also raises the question of how much of the debate surrounding AI is about the perceived value of coding as a technique for qualitative data analysis. In particular, coding is now the basis for such widely used approaches to qualitative research as grounded theory (e.g., Charmaz, 2014), interpretive phenomenological analysis (Smith et al., 2022), and reflexive thematic analysis (Braun & Clarke, 2022). Even with specialized software, such coding requires a great deal of time and effort, so it is entirely possible that the sheer user-friendliness of programs like ChatGPT will produce claims that it makes the analysis process “too easy.” Hence, qualitative researchers who have relied on coding may treat AI as a contradiction to their accustomed way of doing things, rather than as a relief from the time-consuming tedium of coding. Overall, if it can be shown that AI-based analyses produce results that are as good as those from coding at a fraction of the time and expense, then their efficiency cannot be ignored.

The real question, however, is whether AI-based analyses can generate the desired results for these versions of qualitative data analysis. Comparing the three approaches listed above, I think QBA is best suited to replace the more generic approach to locating themes that is outlined in reflexive thematic analysis (Braun & Clarke, 2022). In their six-step process, Steps 1 (familiarization with the data) and 6 (writing up the results) would remain the same, while Steps 3 (detailed coding), 4 (locating initial themes), and 5 (refining final themes) would all be streamlined by the use of AI. It is thus not surprising that there is already a literature on using AI as a basis for reflexive thematic analysis (e.g., Christou, 2024; De Paoli, 2024; Lee et al., 2024; Prescott et al., 2024; Zhang et al., 2023).

QBA would offer similar benefits when applied to grounded theory (e.g., Charmaz, 2014), in terms of reducing the effort involved in open-coding, axial coding, and the final consolidation of codes into conceptual categories. The main difference between applying AI to reflexive thematic analysis and ground theory lies in the latter’s emphasis on producing substantive theories that are more than a list of themes. I believe that AI can also assist in this task, but that goes well beyond the mechanics of coding. In any event, attempts to apply AI to grounded theory are only just beginning (e.g., Tschisgale et al., 2023).

Finally, I have singled out interpretive phenomenological analysis (Smith et al., 2022) as having the least overlap with QBA simply because of its strong basis in phenomenological theory. Although the specific approach to phenomenology in interpretive phenomenological analysis does rely on coding, its version of coding is still theoretically based. How effectively a researcher who was well trained in interpretive phenomenological analysis could make use of AI remains to be seen.

In addition to these practical considerations, the comparison of AI-based analysis and traditional coding also raises the question of whether coding has some theoretical basis that makes it superior to generative AI. At one level, both CAQDAS and programs such as ChatGPT are tools for achieving the crucial goal of interpreting the data. At a more methodological level, the introduction of AI as a replacement for coding also raises broader concerns. In particular, the potential shift to relying on AI requires a consideration of what difference it makes to analyze qualitative data in one way versus another. This points to the need to be reflexive about how and why we analyze our data the way we do.

Beyond the kind of reflexive judgment about one’s data-oriented decisions that I have emphasized throughout, Trena Paulus and her colleagues (Paulus & Lester, 2024; Paulus et al., 2025) argue that further forms of reflexivity are especially important in considering researchers’ choices about the technology they use. In particular, Paulus et al. (2025) distinguish between reflexivity with regard to a researcher’s methods-based choices in a given study and reflexivity with regard to the research practices of a larger discipline. For the first form of reflexivity, they point out that it is not just a matter of reporting why you used a given method and how you did so, but also carefully considering the consequences of those design choices. In the illustrative case presented earlier, I outlined the basis for both my decisions in generating the data (i.e., queries to ChatGPT) and the consequences of those decisions (i.e., my interpretations of the responses from the program). Going forward, the exploratory nature of many applications of AI to qualitative data analysis means that transparency about this kind of within-study reflexivity will be crucial.

The second kind of reflexivity that Paulus et al. (2025) describe is especially important in methodological work such as this article, so that other researcher can see the implications of the proposed method for their own work. As Paulus et al. (2025) state, this involves “moving from exploring the consequences of digital methods on particular study outcomes to engaging the discipline in conversations around how these methods will impact the discipline as a whole” (p. 9). I will argue for two major changes that would result from shifting to AI-based analysis, the first of which is relying on AIs to provide a summary of the data. This is the stage that replaces coding, and it requires a recognition that coding is as much an organizational process as an analytic one. If one of the major purposes of coding is to understand “what is in the data,” then AI-based approaches can be just as effective and far more efficient in accomplishing this regard.

Another major change that I would foresee is toward a much more conversational approach to data analysis in general, and especially to analyses that produce themes. The key point, as demonstrated in the second stage of QBA, is to go beyond merely accepting the AI’s suggestions for themes, and to ask further queries about the bases for and implications of those candidate themes. One of the main strengths of generative AI programs such as ChatGPT is their ability to respond to further prompts with more depth and detail. This provides a powerful set of options for additional reflexive decision making in a way that has no parallels in traditional approaches to qualitative data analysis.

The magnitude of these changes raises the question of whether AI-based methods amount to a full-scale paradigm shift for qualitative data analysis, and here I agree with Friese (forthcoming) that they do not go that far. Following Kuhn (1969), such a paradigm shift would require a larger change in our worldview about the nature and meaning of qualitative analysis, and I do not believe that is the case. Instead, we will continue to pursue the fundamental objective of searching for meaningful interpretations of qualitative data, but we will have new ways of going about that. Rather than a new paradigm, I believe that AI is a new invention that provides innovative ways to accomplish well-established goals.

Ultimately, if QBA and other AI-approaches to qualitative data analysis do gain a substantial foothold, this will be one more example of the larger trend of AI automating crafts that once were done by hand. Hopefully, the successful diffusion of such innovations will be based on their actual effectiveness in accomplishing such tasks. If so, then the present example points to artificial intelligence as a powerful tool for qualitative research.
